# Manganese(III) Acetate-mediated Oxidative Cyclization of α-Methylstyrene and *trans*-Stilbene with β-Ketosulfones

**DOI:** 10.3390/molecules18044293

**Published:** 2013-04-11

**Authors:** Ahlem Bouhlel, Christophe Curti, Clémence Tabelé, Patrice Vanelle

**Affiliations:** Laboratoire de Pharmaco-Chimie Radicalaire, Faculté de Pharmacie, Aix-Marseille Univ, CNRS, Institut de Chimie Radicalaire ICR, UMR 7273, 27 Bd Jean Moulin, CS 30064, 13385 Marseille Cedex 05, France

**Keywords:** manganese(III) acetate, dihydrofuran, radicals

## Abstract

A convenient microwave irradiation protocol was utilized for the synthesis of β-ketosulfones **1**–**5** in good yields. These sulfones reacted with alkenes through a radical oxidative cyclization mediated by Mn(OAc)_3_. Dihydrofurans **6**–**10** were obtained in moderate to good yields starting from 1,1-disubstituted alkenes. Dihydrofurans **11**–**15** were synthesized in moderate yields and unexpected cyclopropanes **16**–**19** were obtained in low yields starting from 1,2-disubstituted alkenes. This protocol offers access to various dihydrofurans which could be tested for their antiparasitic potential.

## 1. Introduction

Manganese(III) acetate has received considerable attention over the past several decades, and remains a useful tool for carbon-carbon bond formation [1,2,3,4]. Its specificity for carbonyl derivatives allows a wide variety of radical synthetic applications, leading to various structures like pyrrolidinones [5], γ-lactones [6,7], tetralins [8,9] or spirocyclic derivatives [10,11,12]. Within our research program directed towards the development of original synthesis methods in medicinal chemistry [13,14,15,16,17], we have explored the radical cyclization of β-ketosulfones [18,19] mediated by manganese(III) acetate in order to synthesize dihydrofurans as potential antiparasitic compounds.

The valuable antileishmanial activities of the functionalized dihydrofurans obtained call for pharmacomodulation starting from new alkene building blocks. Dihydrofuran derivatives are known to have other useful pharmacological properties, such as antibacterial [20,21,22], antifungal [20,21] and anticancer activity [23], as well as being valuable potential intermediates in the synthesis of various substances [24]. A wide variety of methods yielding dihydrofurans and cyclopropanes substituted by a sulfonyl group have been developed [25,26,27,28,29]. We report here the reactivity of two alkenes, α-methylstyrene and *trans*-stilbene, extending previous work on styrene, allylbenzene [30], 1.1-diphenylethylene and derivatives [30,31] and the α,β-ethylenic ketone series [32]. 

## 2. Results and Discussion

**Scheme 1:**
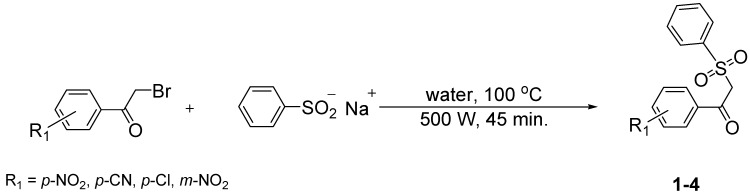
Microwave assisted synthesis of β-ketosulfones **1**–**4**.

*β*-ketosulfones **1-5** were synthesized using previously reported methods [33,34]. As reported in the literature [35,36,37], we observed formation of β-ketosulfones rather than β-ketosulfinate esters. An aqueous solution of sodium benzene sulfinate and the corresponding 2-bromoacetophenone was irradiated at 500 W, 100 °C in a microwave oven for 45 min to give sulfones **1**–**4** in good yields (61–92%) (Scheme 1).

**Scheme 2:**
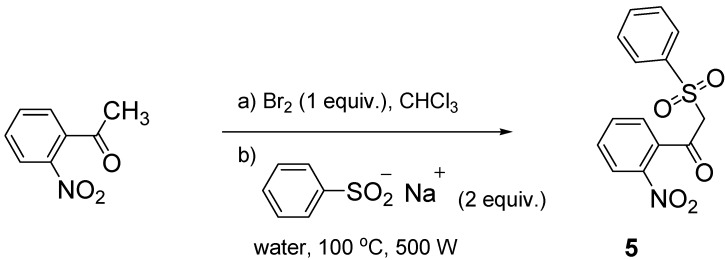
β-ketosulfone **5** microwave assisted synthesis.

*β*-Ketosulfone **5** was synthesized starting with bromination of the corresponding ketone, and substitution with benzene sodium sulfinate without further purification (Scheme 2). The *β*-ketosulfone derivatives thus obtained are presented in Table 1.

**Table 1:** Microwave assisted synthesis of β-ketosulfones.

Entry	Product	Yields ^a^
1	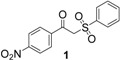	92%
2	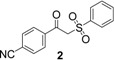	61%
3	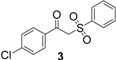	89%
4	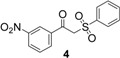	88%
5	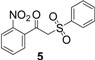	65%

^a^ Yield of isolated product based on the corresponding ketone.

A suspension of manganese(III) acetate in glacial acetic acid was irradiated at 200 W in a microwave oven at 80 °C for 15 min until solubilization. *β*-Ketosulfones **1**–**5** and the corresponding alkenes were added to this solution and the mixture was then irradiated at 200 W, 80 °C for 45 min. 

**Scheme 3:**
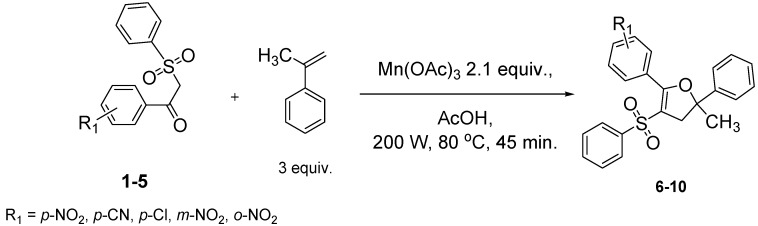
Mn(OAc)_3_ mediated β-ketosulfone reactivity in the α-methylstyrene series.

Using α-methylstyrene as starting alkene, the desired 2,3-dihydrofuran derivatives **6**–**10**, were obtained (Scheme 3) in moderate yields (34–51%, Table 2), with several inseparable secondary products. Attempts were made to increase these yields by adjusting several parameters [Cu(OAc)_2_, more equivalents of alkene, more equivalents of Mn(OAc)_3_] without effect .

**Scheme 4:**
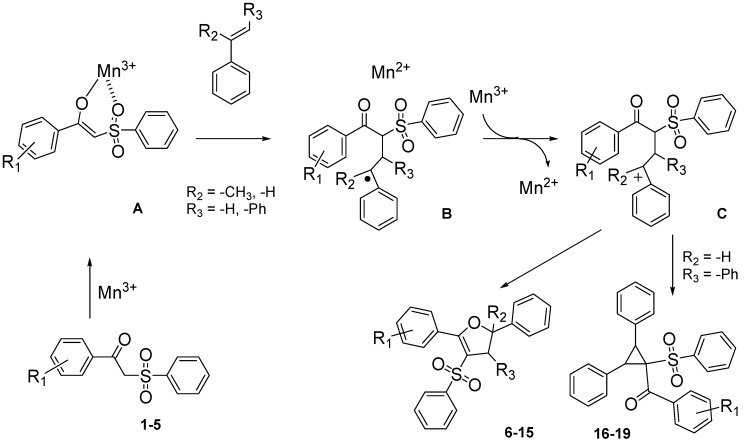
Suggested mechanism of the reaction of β-ketosulfones and Mn(OAc)_3_.

The yield (43%) obtained with 1-(4-nitrophenyl)-2-(phenylsulfonyl)ethanone (**1**) is consistent with previous results [30]. Dihydrofuran was obtained with a lower yield (32%) when the same β-ketosulfone reacted with styrene under the same conditions. Moreover, a better yield (48%) was obtained in the 1,1-diphenylethylenic series. Yields obtained from radical oxidative cyclization of β-ketosulfones directly depend on the stability of the carbon centered radical of intermediate product B (Scheme 4). Starting from α-methylstyrene, this radical stability appears to be intermediate between the stability of the radical from styrene and the radical from 1,1-diphenylethylene.

**Table 2:** Oxidative cyclizations mediated by Mn(OAc)_3_.

Entry	β-ketosulfone/alkene	Product/Yields ^a^	
1	**1**/α-methylstyrene	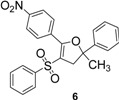 /43%	
2	**2**/α-methylstyrene	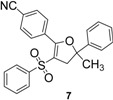 /34%	
3	**3**/α-methylstyrene	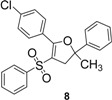 /35%	
4	**4**/α-methylstyrene	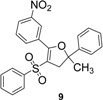 /51%	
5	**5**/α-methylstyrene	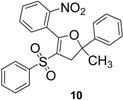 /45%	
6	**1**/ *trans*-stilbene	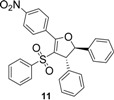 /21%	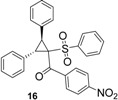 /12%
7	**2**/ *trans*-stilbene	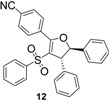 /13%	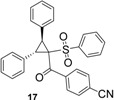 /10%
8	**3**/ *trans*-stilbene	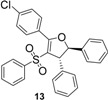 /12%	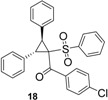 /5%
9	**4**/ *trans*-stilbene	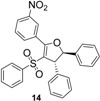 /25%	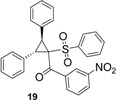 /13%
10	**5**/ *trans*-stilbene	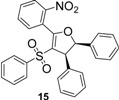 /23%	

^a^ Yield of isolated product based on the corresponding sulfone.

**Scheme 5:**
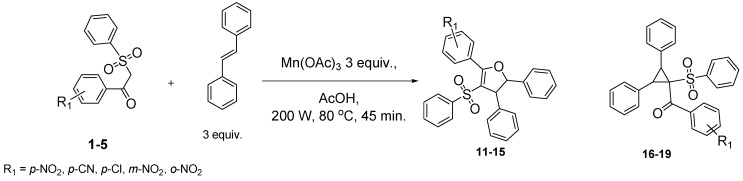
Mn(OAc)_3_ mediated reactivity of β-ketosulfones in the *trans*-stilbene series.

Using *trans*-stilbene as starting alkene, 2,3-dihydrofuran derivatives **11**–**15** were obtained (Scheme 5) in moderate yields (12–25%, Table 2) and original cyclopropanes **16**–**19** were also obtained in low yields (5–13%). With 1-(4-nitrophenyl)-2-(phenylsulfonyl)ethanone (**1**) 24% of the starting material was recovered, along with several inseparable products. Dihydrofuran and cyclopropane yields were slightly increased by using 3 equiv. of Mn(OAc)_3_, but not by using Cu(OAc)_2_ or more equivalents of alkene.

From analysis of the ^1^H-NMR spectra of synthesized products **11**–**19** and comparison with the previously reported results, we conclude that dihydrofuran and cyclopropane derivatives were obtained diastereoselectively from β-ketosulfones **1**–**4** as the corresponding *trans* isomers.

Both protons of the dihydrofuran rings in **11**–**14** display two doublets at mean values of 5.50 and 4.50 ppm, with a vicinal coupling constant *J* between 5.0 and 5.6 Hz. For *cis*-2,3-dihydrofuran, the vicinal coupling constant of the two methine protons proved to be *J* = 7–10 Hz, while for *trans*-2,3-dihydrofuran the vicinal coupling constant *J* = 4–7 Hz [38]. Moreover, these results agree with previous studies reporting *trans*-dihydrofuran diastereoselective synthesis *via* Mn(OAc)_3_ mediated radical oxidative cyclizations starting from 1,2-disubstituted alkenes and β-ketonitriles [39,40] or β-ketoesters [41]. One of these studies [40] showed that the stereochemistry of Mn(OAc)_3_ mediated oxidative cyclizations is not influenced by the stereochemistry of the starting alkene (*cis* or *trans*-stilbene).

**Figure 1:**
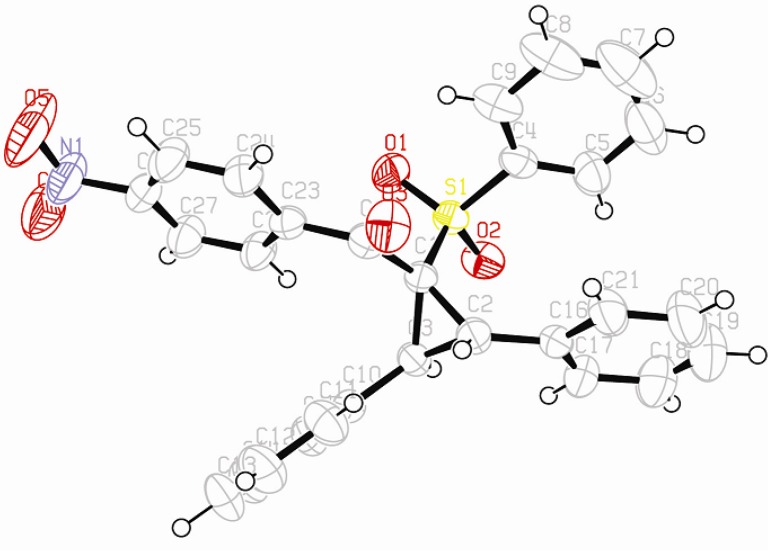
ORTEP view of compound **16**.

To our knowledge, few studies have reported intramolecular cyclopropanation under Mn(OAc)_3_ reactivity [42,43,44,45], and only one reported intermolecular reactions between oxabenzonorbornadiene and dimedone [46]. As Mn(OAc)_3_-mediated intermolecular cyclopropanation with *trans*-stilbene or a similar 1,2-disubstituted alkene has never been reported, the structure of cyclopropane **16** was established by X-ray diffraction analysis (Figure 1). As specified in previous studies [44], Mn(OAc)_3_ mediated cyclopropanation should come from the cation C (Schem 4), which reacted rapidly with the enolic form of β-ketosulfone to close the cyclopropane ring.

β-ketosulfone **5** led to *cis*-dihydrofuran **15** with vicinal coupling constant *J* = 8.0 Hz. *o*-Nitro substitution appears to have reversed the diastereoselectivity and inhibited the cyclopropanation, probably through the stereoelectronic effects of the nitro group during the final cyclization step. Further research is in progress to explore this original mechanism.

## 3. Experimental

### 3.1. General

Microwave-assisted reactions were performed in a multimode microwave oven (ETHOS Synth Lab Station, Ethos start, Milestone Inc., Rockford, IL, USA). Melting points were determined with a B-540 Büchi melting point apparatus. ^1^H-NMR (200 MHz) and ^13^C-NMR (50 MHz) spectra were recorded on a Bruker ARX 200 spectrometer in CDCl_3_ or D_2_O at the Service Interuniversitaire de RMN de la Faculté de Pharmacie de Marseille. The ^1^H chemical shifts were reported as parts per million downfield from tetramethylsilane (Me_4_Si), and the ^13^C chemical shifts were referenced to the solvent peaks: CDCl_3_ (76.9 ppm) or DMSO-*d_6_* (39.6 ppm). Absorptions were reported with the following notations: s, singlet; bs, broad singlet; d, doublet; t, triplet; q, quartet; m, a more complex multiplet or overlapping multiplets. Elemental analysis and mass spectra, run on an API-QqToF mass spectrometer, were carried out at the Spectropole de la Faculté des Sciences site Saint-Jérôme. Mass spectra, run on a MicrOToF Q mass spectrometer, were carried out at the Plateforme Protéomique Innovation Technologique Timone (PIT2) UMR 911 Faculté de Pharmacie. The following adsorbent was used for flash column chromatography: silica gel 60 (Merck, particle size 0.040–0.063 nm, 70–230 mesh ASTM). TLC were performed on 5 cm × 10 cm aluminium plates coated with silica gel 60 F-254 (Merck) in an appropriate solvent.

### 3.2. General Procedure for the Synthesis of β-Ketosulfones **1**–**5**

*Method A, starting from α-bromoacetophenone*: To a solution of sodium benzene sulfinate (4.1 mmol, 2 equiv.) in water (30 mL), an ethanolic solution of the corresponding acetophenone (2.05 mmol, 1 equiv.) was added. The reaction mixture was heated under reflux in a microwave oven under irradiation (500 W, 100 °C) during 45 min. The precipitate thus formed was filtered and crystallized from the appropriate solvent.

*Method B, starting from acetophenone*: To a solution of 1-(2-nitrophenyl)ethanone (3 g, 18.00 mmol, 1 equiv.) in chloroform (15 mL), a bromine (2.9 g, 18.00 mmol, 1 equiv.) solution in chloroform (7 mL) was slowly added. After 1 h at rt, the reaction mixture was washed 3 times with a saturated solution of sodium thiosulfate, and concentrated under vacuum. A solution of sodium benzene sulfinate (6 g, 36.00 mmol, 2 equiv.) in water (30 mL) was added. The reaction mixture was heated under reflux in a microwave oven under irradiation (500 W, 100 °C) during 45 min. The precipitate thus formed was filtered and crystallized from the appropriate solvent.

*1-(4-Nitrophenyl)-2-(phenylsulfonyl)ethanone* (**1**). Colorless crystals, mp 138 °C (ethanol) (Lit. [47,48]: 136–138 °C), yield 92%. ^1^H-NMR (CDCl_3_), δ: 8.34 (d, *J* = 8.9 Hz, 2H), 8.15 (d, *J* = 8.9 Hz, 2H), 7.88 (d, *J* = 7.2 Hz, 2H), 7.64 (m, 3H), 4.77 (s, 2H). ^13^C-NMR (CDCl_3_), δ: 64.0 (CH_2_), 124.0 (2CH), 128.5 (2CH), 129.4 (2CH), 130.5 (2CH), 134.6 (CH), 138.4 (C), 140.0 (C), 186.8 (C=O), 1C not observed in these conditions.

*4-(2-(Phenylsulfonyl)acetyl)benzonitrile* (**2**) [19]. White solid, mp 150–151 °C (isopropanol); yield 61%. ^1^H-NMR (CDCl_3_), δ: 4.74 (s, 2H, CH_2_), 7.53–7.72 (m, 3H, 3CH), 7.79 (d, *J* = 8.3, 2H, 2CH), 7.85–7.89 (m, 2H, 2CH), 8.07 (d, *J* = 8.3, 2H, 2CH). ^13^C-NMR (CDCl_3_), δ: 63.7 (CH_2_), 117.5 (2C), 128.5 (2CH), 129.4 (2CH), 129.7 (2CH), 132.6 (2CH), 134.6(CH), 138.4 (C), 138.5 (C), 197.0 (C).

*1-(4-Chlorophenyl)-2-(phenylsulfonyl)ethanone* (**3**). White solid; mp 135–137 °C (isopropanol) (Lit. [49]: 133–135 °C); yield 89%. ^1^H-NMR (CDCl_3_) *δ*_H_ 4.70 (s, 2H, CH_2_), 7.44–7.73 (m, 5H, 5CH), 7.86–7.92 (m, 4H, 4CH). ^13^C-NMR (CDCl_3_) *δ*_C_ 63.6 (CH_2_), 128.5 (2CH), 129.2 (2CH), 129.3 (2CH), 130.7 (2CH), 134.0 (C), 134.4 (CH), 138.5 (C), 141.1 (C), 186.8 (C).

*1-(3-Nitrophenyl)-2-(phenylsulfonyl)ethanone* (**4**). White solid; mp 127–129 °C (isopropanol) (Lit. [50]: 128–129 °C); yield 88%. ^1^H-NMR (CDCl_3_) *δ*_H_ 4.79 (s, 2H, CH_2_), 7.53–7.61 (m, 2H, 2CH), 7.67–7.77 (m, 2H, 2CH), 7.87–7.91 (m, 2H, 2CH), 8.34 (ddd, *J_1_* = 1.1, *J_2_* = 1.5, *J_3_* = 7.8, 1H, CH), 8.48 (ddd, *J_1_* = 1.1, *J_2_* = 2.1, *J_3_* = 8.2, 1H, CH), 8.73–8.75 (m, 1H, CH). ^13^C-NMR (CDCl_3_) *δ*_C_ 63.7 (CH_2_), 124.1 (CH), 128.4 (CH), 128.5 (2CH), 129.4 (2CH), 130.2 (CH), 134.6 (CH), 134.8 (CH), 136.8 (C), 138.3 (C), 148.5 (C), 186.2 (C). 

*1-(2-Nitrophenyl)-2-(phenylsulfonyl)ethanone* (**5**). Yellow oil; yield 65%. ^1^H-NMR (CDCl_3_) *δ*_H_ 4.61 (s, 2H, CH_2_), 7.54–7.70 (m, 5H, 5CH), 7.73–7.84 (m, 1H, CH), 7.88–7.92 (m, 2H, 2CH), 8.12–8.16 (m, 1H, CH). ^13^C-NMR (CDCl_3_) *δ*_C_ 66.6 (CH_2_), 124.2 (CH), 128.3 (2CH), 129.0 (CH), 129.4 (2CH), 131.5 (CH), 134.4 (CH), 135.0 (CH), 136.2 (C), 138.7 (C), 145.1 (C), 189.8 (C). HMRS (ESI): *m/z* calcd for C_14_H_11_NO_5_S M+Na^+^: 328.0250. Found: 328.02471. 

### 3.3. General Procedure for the Synthesis of Dihydrofurans **6**–**10**

A suspension of manganese(III) acetate dihydrate (1.84 g, 6.87 mmol, 2.1 equiv.) in glacial acetic acid (30 mL) was heated under microwave irradiation (200 W, 80 °C) for 15 min, until dissolution. Then, the reaction mixture was cooled down to 50 °C, and a solution of the corresponding β-keto-sulfone (3.27 mmol, 1 equiv.) and α-methylstyrene (1.16 g, 9.81 mmol, 3 equiv.) in acetic acid (5 mL) was added. The mixture was heated under microwave irradiation (200 W, 80 °C) for 45 min. The reaction mixture was poured into 200 mL of cold water, and extracted with chloroform (3 × 40 mL). The organic extracts were collected and washed with saturated aqueous NaHCO_3_ (3 × 40 mL) and dried (MgSO_4_). Solvent evaporation was followed by column chromatography (petroleum ether/chloroform/diethyl ether 5/4.5/0.5), and the product obtained was recrystallized from isopropanol.

*2-Methyl-5-(4-nitrophenyl)-2-phenyl-4-(phenylsulfonyl)-2,3-dihydrofuran* (**6**). White solid; mp 144–145 °C; yield 43%. ^1^H-NMR (CDCl_3_) *δ*_H_ 1.74 (s, 3H, CH_3_), 3.26 (d, *J* = 14.8, 1H, CH_2_), 3.38 (d, *J* = 14.8, 1H, CH_2_), 7.29–7.58 (m, 8H, 8CH), 7.70–7.74 (m, 2H, 2CH), 7.94 (d, *J* = 8.9, 2H, 2CH), 8.30 (d, *J* = 8.9, 2H, 2CH). ^13^C-NMR (CDCl_3_) *δ*_C_ 29.1 (CH_3_), 45.8 (CH_2_), 89.7 (C), 112.6 (C), 123.0 (2CH), 124.0 (2CH), 126.9 (2CH), 128.0 (CH), 128.7 (2CH), 129.2 (2CH), 130.8 (2CH), 133.2 (CH), 134.7 (C), 141.1 (C), 144.3 (C), 149.0 (C), 159.6 (C). HMRS (ESI): *m/z* calcd for C_23_H_19_NO_5_S M+H^+^: 422.1057. Found: 422.1058.

*4-(5-Methyl-5-phenyl-3-(phenylsulfonyl)-4,5-dihydrofuran-2-yl)benzonitrile* (**7**). White solid; mp 137–138 °C; yield 34%. ^1^H-NMR (CDCl_3_) *δ*_H_ 1.73 (s, 3H, CH_3_), 3.25 (d, *J* = 14.7, 1H, CH_2_), 3.37 (d, *J* = 14.7, 1H, CH_2_), 7.28–7.61 (m, 9H, 9CH), 7.68–7.76 (m, 3H, 3CH), 7.89 (d, *J* = 8.7, 2H, 2CH). ^13^C-NMR (CDCl_3_) *δ*_C_ 29.1 (CH_3_), 45.8 (CH_2_), 89.5 (C), 112.2 (C), 114.4 (C), 118.2 (C), 124.0 (2CH), 126.8 (2CH), 127.9 (CH), 128.7 (2CH), 129.1 (2CH), 130.3 (2CH), 131.6 (2CH), 132.9 (C), 133.2 (CH), 141.1 (C), 144.4 (C), 159.9 (C). HMRS (ESI): *m/z* calcd for C_24_H_19_NO_3_S M+H^+^: 402.1158. Found: 402.1157.

*5-(4-Chlorophenyl)-2-methyl-2-phenyl-4-(phenylsulfonyl)-2,3-dihydrofuran* (**8**). White solid; mp 107–109 °C; yield 35%. ^1^H-NMR (CDCl_3_) *δ*_H_ 1.71 (s, 3H, CH_3_), 3.25 (d, *J* = 14.5, 1H, CH_2_), 3.36 (d, *J* = 14.5, 1H, CH_2_), 7.29–7.54 (m, 10H, 10CH), 7.68–7.77 (m, 4H, 4CH). ^13^C-NMR (CDCl_3_) *δ*_C_ 29.0 (CH_3_), 45.9 (CH_2_), 88.8 (C), 110.2 (C), 124.1 (2CH), 126.7 (2CH), 127.0 (C), 127.7 (CH), 128.2 (2CH), 128.6 (2CH), 129.0 (2CH), 131.0 (2CH), 132.9 (CH), 137.2 (C), 141.6 (C), 144.7 (C), 161.2 (C). HMRS (ESI): *m/z* calcd for C_23_H_19_ClO_3_S M+H^+^: 411.0816. Found: 411.0813.

*2-Methyl-5-(3-nitrophenyl)-2-phenyl-4-(phenylsulfonyl)-2,3-dihydrofuran* (**9**). Yellow oil; yield 51%. ^1^H-NMR (CDCl_3_) *δ*_H_ 1.75 (s, 3H, CH_3_), 3.27 (d, *J* = 14.7, 1H, CH_2_), 3.41 (d, *J* = 14.7, 1H, CH_2_), 7.24–7.79 (m, 11H, 11CH), 8.14–8.19 (m, 1H, CH), 8.31–8.37 (m, 1H, CH), 8.55–8.57 (m, 1H, CH). ^13^C-NMR (CDCl_3_) *δ*_C_ 29.1 (CH_3_), 45.7 (CH_2_), 89.6 (C), 112.0 (C), 124.0 (2CH), 124.4 (CH), 125.5 (CH), 126.9 (2CH), 127.8 (CH), 128.4 (C), 128.7 (2CH), 129.0 (CH), 129.2 (2CH), 130.1 (C), 133.2 (CH), 135.8 (CH), 141.1 (C), 144.3 (C), 159.3 (C). HMRS (ESI): *m/z* calcd for C_23_H_19_NO_5_S M+Na^+^: 444.0876. Found: 444.0880. 

*2-Methyl-5-(2-nitrophenyl)-2-phenyl-4-(phenylsulfonyl)-2,3-dihydrofuran* (**10**). White solid; mp 104–105 °C; yield 45%. ^1^H-NMR (CDCl_3_) *δ*_H_ 1.75 (s, 3H, CH_3_), 3.12 (d, *J* = 14.2, 1H, CH_2_), 3.37 (d, *J* = 14.2, 1H, CH_2_), 7.31–7.36 (m, 4H, 4CH), 7.43–7.80 (m, 9H, 9CH), 8.15–8.20 (m, 1H, CH). ^13^C-NMR (CDCl_3_) *δ*_C_ 28.7 (CH_3_), 44.8 (CH_2_), 91.0 (C), 111.1 (C), 124.3 (2CH), 124.5 (CH), 124.8 (C), 127.1 (2CH), 127.8 (CH), 128.6 (2CH), 129.1 (2CH), 131.2 (CH), 133.0 (CH), 133.1 (2CH), 140.7 (C), 144.5 (C), 147.5 (C), 159.6 (C). HMRS (ESI): *m/z* calcd for C_23_H_19_NO_5_S M+NH_4_^+^: 439.1322. Found: 439.1326.

### 3.4. General Procedure for the Synthesis of Dihydrofurans **11**–**15** and Cyclopropanes **16**–**19**

A suspension of manganese(III) acetate dihydrate (2.63 g, 9.81 mmol, 3 equiv.) in glacial acetic acid (30 mL) was heated under microwave irradiation (200 W, 80 °C) for 15 min, until dissolution. Then, the reaction mixture was cooled down to 50 °C, and a solution of the corresponding β-ketosulfone (3.27 mmol, 1 equiv.) and *trans*-stilbene (1.77 g, 9.81 mmol, 3 equiv.) in acetic acid (5 mL) was added. The mixture was heated under microwave irradiation (200 W, 80 °C) for 45 min. The reaction mixture was poured into 200 mL of cold water, and extracted with chloroform (3 × 40 mL). The organic extracts were collected and washed with saturated aqueous NaHCO_3_ (3 × 40 mL) and dried (MgSO_4_). Solvent evaporation was followed by column chromatography (petroleum ether/chloroform/diethyl ether 5/4.5/0.5), and the products obtained were recrystallized from isopropanol.

*5-(4-Nitrophenyl)-2,3-diphenyl-4-(phenylsulfonyl)-2,3-dihydrofuran* (**11**). Yellow solid; mp 178–180 °C; yield 21%. ^1^H-NMR (CDCl_3_) *δ*_H_ 4.50 (d, *J* = 5.6, 1H, CH), 5.56 (d, *J* = 5.6, 1H, CH), 7.14–7.42 (m, 15H, 15CH), 8.03 (d, *J* = 8.8, 2H, 2CH), 8.32 (d, *J* = 8.8, 2H, 2CH). ^13^C-NMR (CDCl_3_) *δ*_C_ 59.2 (CH), 92.0 (CH), 117.5 (C), 123.1 (2CH), 125.1 (2CH), 127.1 (2CH), 127.8 (2CH), 127.9 (CH), 128.6 (2CH), 129.0 (2CH), 129.1 (2CH), 129.9 (CH), 132.8 (CH), 134.7 (C), 139.6 (C), 140.3 (C), 141.4 (C), 149.2 (C), 161.9 (C). HMRS (ESI): *m/z* calcd for C_28_H_21_NO_5_S M+Na^+^: 506.1033. Found: 506.1032.

*4-(4,5-Diphenyl-3-(phenylsulfonyl)-4,5-dihydrofuran-2-yl)benzonitrile* (**12**). Yellow solid; mp 182–184 °C; yield 13%. ^1^H-NMR (CDCl_3_) *δ*_H_ 4.48 (d, *J* = 5.5, 1H, CH), 5.54 (d, *J* = 5.5, 1H, CH), 7.13–7.30 (m, 8H, 8CH), 7.36–7.45 (m, 7H, 7CH), 7.76 (d, *J* = 8.6, 2H, 2CH), 7.98 (d, *J* = 8.6, 2H, 2CH). ^13^C-NMR (CDCl_3_) *δ*_C_ 59.2 (CH), 91.8 (CH), 114.7 (C), 117.1 (C), 118.1 (C), 125.1 (2CH), 127.1 (2CH), 127.8 (2CH), 128.6 (2CH), 129.1 (2CH), 130.4 (2CH), 131.7 (2CH), 132.8 (CH), 132.9 (C), 139.7 (C), 140.5 (C), 141.4 (C), 162.2 (C). HMRS (ESI): *m/z* calcd for C_29_H_21_NO_3_S M+Na^+^: 486.1134. Found: 486.1131.

*5-(4-Chlorophenyl)-2,3-diphenyl-4-(phenylsulfonyl)-2,3-dihydrofuran* (**13**). White solid; mp 170–171 °C; yield 12%. ^1^H-NMR (CDCl_3_) *δ*_H_ 4.46 (d, *J* = 5.0, 1H, CH), 5.51 (d, *J* = 5.0, 1H, CH), 7.17–7.41 (m, 15H, 15CH), 7.45 (d, *J* = 8.5, 2H, 2CH), 7.82 (d, *J* = 8.5, 2H, 2CH). ^13^C-NMR (CDCl_3_) *δ*_C_ 59.3 (CH), 91.3 (CH), 115.3 (C), 125.0 (2CH), 126.9 (C), 127.0 (2CH), 127.7 (CH), 127.8 (2CH), 128.4 (2CH), 128.5 (2CH), 128.8 (CH), 129.0 (4CH), 131.1 (2CH), 132.5 (CH), 137.6 (C), 140.1 (C), 141.1 (C), 141.9 (C), 163.5 (C). HMRS (ESI): *m/z* calcd for C_28_H_21_ClO_3_S M+Na^+^: 495.0792. Found: 495.07884.

*5-(3-Nitrophenyl)-2,3-diphenyl-4-(phenylsulfonyl)-2,3-dihydrofuran* (**14**). White solid; mp 147–148 °C; yield 25%. ^1^H-NMR (CDCl_3_) *δ*_H_ 4.56 (d, *J* = 5.6, 1H, CH), 5.57 (d, *J* = 5.6, 1H, CH), 7.22–7.32 (m, 11H, 11CH), 7.38–7.42 (m, 4H, 4CH), 7.65–7.73 (m, 1H, CH), 8.23–8.26 (m, 1H, CH), 8.36–8.42 (m, 1H, CH), 8.63–8.65 (m, 1H, CH). ^13^C-NMR (CDCl_3_) *δ*_C_ 59.1 (CH), 92.0 (CH), 117.0 (C), 124.7 (CH), 125.2 (2CH), 125.8 (CH), 127.2 (2CH), 127.9 (2CH), 128.7 (2CH), 129.1 (2CH), 129.2 (2CH), 129.3 (CH), 130.2 (C), 132.8 (CH), 135.8 (CH), 139.6 (C), 140.5 (C), 141.5 (C), 147.7 (C), 161.7 (C). HMRS (ESI): *m/z* calcd for C_28_H_21_NO_5_S M+Na^+^: 506.1032. Found: 506.1034.

*5-(2-Nitrophenyl)-2,3-diphenyl-4-(phenylsulfonyl)-2,3-dihydrofuran* (**15**). White solid; mp 155–156 °C; yield 23%. ^1^H-NMR (CDCl_3_) *δ*_H_ 4.62 (d, *J* = 8.0, 1H, CH), 5.66 (d, *J* = 8.0, 1H, CH), 7.08–7.20 (m, 9H, 9CH), 7.28–7.39 (m, 6H, 6CH), 7.67–7.87 (m, 3H, 3CH), 8.24–8.18 (m, 1H, CH). ^13^C-NMR (CDCl_3_) *δ*_C_ 58.7 (CH), 93.8 (CH), 115.8 (C), 124.7 (CH), 125.0 (C), 125.8 (2CH), 127.2 (2CH), 127.6 (2CH), 128.5 (2CH), 128.6 (2CH), 128.8 (2CH), 128.9 (CH), 129.0 (2CH), 131.4 (CH), 132.5 (CH), 133.3 (CH), 133.4 (CH), 139.3 (C), 139.4 (C), 141.3 (C), 147.4 (C), 162.0 (C). HMRS (ESI): *m/z* calcd for C_28_H_21_NO_5_S M+NH_4_^+^: 501.1479. Found: 501.1482.

*(2,3-Diphenyl-1-(phenylsulfonyl)cyclopropyl)(4-nitrophenyl)methanone* (**16**). White solid; mp 223 °C; yield 12%. ^1^H-NMR (CDCl_3_) *δ*_H_ 3.70 (d, *J* = 9.4, 1H, CH), 4.45 (d, *J* = 9.4, 1H, CH), 6.94–6.99 (m, 2H, 2CH), 7.13–7.24 (m, 12H, 12CH), 7.46–7.53 (m, 1H, CH), 7.97 (d, *J* = 9.0, 2H, 2CH), 8.07 (d, *J* = 9.0, 2H, 2CH). ^13^C-NMR (CDCl_3_) *δ*_C_ 33.7 (CH), 34.0 (CH), 63.1 (C), 122.7 (2CH), 127.5 (2CH), 127.7 (CH), 128.0 (2CH), 128.1 (2CH), 128.4 (CH), 128.5 (2CH), 128.9 (2CH), 129.9 (2CH), 131.7 (2CH), 133.4 (CH), 138.7 (C), 140.3 (C), 150.1 (C), 190.8 (C). Anal. Calcd for C_28_H_21_NO_5_S: C, 69.55; H, 4.38; N, 2.90. Found: C, 69.48; H, 4.46; N, 2.94.

*4-(2,3-Diphenyl-1-(phenylsulfonyl)cyclopropanecarbonyl)benzonitrile* (**17**). White solid; mp 225–227 °C; yield 10%. ^1^H-NMR (CDCl_3_) *δ*_H_ 3.67 (d, *J* = 9.4, 1H, CH), 4.42 (d, *J* = 9.4, 1H, CH), 6.94–6.99 (m, 2H, 2CH), 7.13–7.28 (m, 12H, 12CH), 7.45–7.51 (m, 1H, CH), 7.52 (d, *J* = 8.4, 2H, 2CH), 7.92 (d, *J* = 8.4, 2H, 2CH). ^13^C-NMR (CDCl_3_) *δ*_C_ 33.6 (CH), 33.9 (CH), 62.8 (C), 116.3 (C), 117.9 (C), 127.5 (2CH), 127.7 (CH), 128.0 (2CH), 128.1 (2CH), 128.3 (CH), 128.4 (2CH), 128.9 (2CH), 129.9 (2CH), 130.2 (C), 131.0 (2CH), 131.4 (2CH), 133.2 (C), 133.4 (CH), 138.6 (C), 138.8 (C), 190.9 (C). HMRS (ESI): *m/z* calcd for C_29_H_21_NO_3_S M+Na^+^: 486.1134. Found: 486.1133. 

*(4-Chlorophenyl)(2,3-diphenyl-1-(phenylsulfonyl)cyclopropyl)methanone* (**18**). White solid; mp 97–98 °C; yield 5%. ^1^H-NMR (CDCl_3_) *δ*_H_ 3.67 (d, *J* = 9.4, 1H, CH), 4.38 (d, *J* = 9.4, 1H, CH), 6.99–7.03 (m, 1H, CH), 7.13–7.24 (m, 4H, 4CH), 7.42–7.53 (m, 8H, 8CH), 7.61–7.68 (m, 2H, 2CH), 7.76–7.87 (m, 2H, 2CH), 7.96 (d, *J* = 8.5, 2H, 2CH). ^13^C-NMR (CDCl_3_) *δ*_C_ 33.7 (CH), 33.8 (CH), 63.0 (C), 121.5 (2CH), 127.5 (CH), 128.0 (2CH), 128.4 (C), 128.5 (2CH), 128.7 (CH), 128.9 (2CH), 129.0 (2CH), 129.9 (2CH), 130.7 (2CH), 132.2 (CH), 134.7 (C), 136.5 (C), 139.2 (C), 145.3 (2CH), 189.2 (C). 1C not observed in these conditions. HMRS (ESI): *m/z* calcd for C_28_H_21_ClO_3_S M+Na^+^: 495.0792. Found: 495.0792. 

*(2,3-Diphenyl-1-(phenylsulfonyl)cyclopropyl)(3-nitrophenyl)methanone* (**19**). Yellow solid; mp 116–118 °C; yield 13%. ^1^H-NMR (CDCl_3_) *δ*_H_ 3.71 (d, *J* = 9.3, 1H, CH), 4.48 (d, *J* = 9.3, 1H, CH), 6.94–7.25 (m, 8H, 8CH), 7.39–7.95 (m, 8H, 8CH), 8.13–8.48 (m, 2H, 2CH), 8.63–8.86 (m, 1H, CH). ^13^C-NMR (CDCl_3_) *δ*_C_ 33.3 (CH), 33.7 (CH), 62.7 (C), 120.6 (CH), 123.3 (CH), 127.1 (CH), 127.5 (2CH), 127.7 (C), 128.1 (2CH), 128.3 (C), 128.5 (CH), 128.7 (2CH), 128.9 (CH), 129.1 (2CH), 129.9 (2CH), 131.2 (CH), 132.9 (C), 134.1 (CH), 134.3 (C), 139.5 (C), 190.0 (C). HMRS (ESI): *m/z* calcd for C_28_H_21_NO_5_S M+NH_4_^+^: 501.1479. Found: 501.1483. 

### 3.5. X-ray Structure Determination of Compounds **16**

Crystal data for compound **16**: C_28_H_21_NO_5_S, *M* = 483.52, monoclinic, *a* = 13.6866(5) Å, *b* = 10.2670(4) Å, *c* = 17.8561(9) Å, *α* = 90°, *β* = 102.777(2)°, *γ* = 90°, *V* = 2447.01(18) Å^3^, *T* = 293(2) K, space group *P*21*/c*, *Z* = 4, *μ*(MoKα) = 0.172 mm^−1^, 18832 reflections measured, 5869 independent reflections (*R_int_* = 0.145). The final *R_1_* values were 0.1086 (*I* > 2*σ*(*I*)). The final *wR*(*F*^2^) values were 0.1595 (*I* > 2*σ*(*I*)). The final *R_1_* values were 0.2171 (all data). The final *wR*(*F*^2^) values were 0.2003 (all data). The goodness of fit on *F*^2^ was 1.094. Crystallographic data (excluding structure factors) for the structures **16** have been deposited with the Cambridge Crystallographic Data Centre (CCDC) under the number 918793. Copies of the data can be obtained, free of charge, on application to CCDC, 12 Union Road, Cambridge CB2 1EZ, UK, (fax: +44-(0)1223-336033 or e-mail: deposit@ccdc.cam.ac.uk).

## 4. Conclusions

Thanks to the radical reactivity of Mn(OAc)_3_, we synthesized 10 new functionalized dihydrofurans, which could offer antiparasitic activities. Starting from *trans*-stilbene alkene, a diastereoselectivity reversion of dihydrofuran was observed with *ortho*-substituted β-ketosulfones. Moreover, four original cyclopropanes were obtained in low yields. This original cyclopropanation reaction could be a very valuable tool in organic synthesis and additional research seeking to enhance yields is currently in progress.
